# Draft Genome Sequences of Nine *Streptococcus suis* Strains Isolated in the United States

**DOI:** 10.1128/genomeA.01301-15

**Published:** 2015-11-05

**Authors:** Samantha J. Hau, Darrell O. Bayles, David P. Alt, Susan L. Brockmeier, Timothy S. Frana, Tracy L. Nicholson

**Affiliations:** a Department of Veterinary Diagnostic and Production Animal Medicine, College of Veterinary Medicine, Iowa State University, Ames, Iowa, USA; b National Animal Disease Center, Agricultural Research Service, USDA, Ames, Iowa, USA

## Abstract

*Streptococcus suis* is a swine pathogen responsible for economic losses to the pig industry worldwide. Additionally, it is a zoonotic agent that can cause severe infections in those in close contact with infected pigs and/or who consume uncooked or undercooked pork products. Here, we report nine draft genome sequences of *S. suis*.

## GENOME ANNOUNCEMENT

The Gram-positive bacterium *Streptococcus suis* asymptomatically colonizes the upper respiratory tract, such as the tonsils and nasal cavity of pigs. *S. suis* can also cause respiratory disease and serious systemic diseases, such as arthritis, septicemia, meningitis, endocarditis, and even sudden death (1, 2). For these reasons, *S. suis* is responsible for significant economic losses to the pig industry worldwide. Zoonotic *S. suis* infections can occur and are thought to be preventable and acquired from infected pigs, either due to occupational exposure or by handling and/or consuming undercooked pork products. The prevalence of human *S. suis* infections is highest in East and Southeast Asia (3–7). Two large outbreaks occurred in China in 1998 and 2005, resulting in 229 human cases with 52 deaths reported, which caused a serious public health concern (8, 9). Thirty-five serotypes of *S. suis* have been identified based on antigenic differences in their capsular polysaccharide; however, only a limited number are responsible for infections in pigs, including serotypes 1 to 9 and 14 (1). Serotype 2 is considered to be the most pathogenic and is the most frequently isolated serotype from both diseased swine and humans (10). Despite a significant impact on swine health, economic losses incurred by the swine industry, and its zoonotic potential, the virulence mechanisms of *S. suis* are poorly understood.

We obtained draft genome sequences of 9 *S. suis* strains to identify the genetic diversity among currently circulating strains within the United States. We obtained and sequenced the genomes of 8 *S. suis* strains (ISU2414, ISU2514, ISU2614, ISU2714, ISU2812, ISU2912, ISU1606, and ISU2660) isolated from pigs exhibiting clinical disease and 1 *S. suis* strain (SRD478) isolated from the nasal cavity of an asymptomatic pig. Genomic DNA sequencing libraries were prepared according to the manufacturer’s directions. Briefly, genomic DNA was prepared using the High Pure PCR template preparation kit (Roche Applied Science, Indianapolis, IN), according to the manufacturer’s directions. The Nextera XT DNA sample preparation and index kits (Illumina, San Diego, CA) were used to convert genomic DNA into indexed libraries suitable for sequencing. The indexed libraries were pooled and sequenced on the Illumina MiSeq platform (Illumina, Inc., San Diego, CA, USA) using the MiSeq version 2 500-cycle reagent kit yielding 2 × 250-bp paired-end reads.

Draft *de novo* genome assemblies were generated using MIRA version 4.0.2 (11). The assembled genomes had the following average coverages: ISU2414, 73×; ISU2514, 72×; ISU2614, 64×; ISU2714, 54×; ISU2812, 57×; ISU2912, 25×; ISU1606, 80×; ISU2660, 72×; and SRD478, 26×. After assembly, contigs were filtered to include only those contigs with two-thirds or more of the average coverage level for the strain and a length >1,500 bp. In addition, if the assembler indicated there was evidence that a contig was part of a repetitive element, we required the contig to be >2,000 bp to be included in the assembly.

### Nucleotide sequence accession numbers.

This whole-genome shotgun project for these isolates has been deposited in DDBJ/ENA/GenBank under the accession numbers ISU2414, LDOG00000000; ISU2514, LDOH00000000; ISU2614, LDOI00000000; ISU2714, LDOJ00000000; ISU2812, LDOK00000000; ISU2912, LDOL00000000; ISU1606, LDOM00000000; ISU2660, LDON00000000; and SRD478, LGKK00000000.
